# Components of Metabolic Syndrome and the Risk of Disability among the Elderly Population

**DOI:** 10.1038/srep22750

**Published:** 2016-03-07

**Authors:** Fang-Yih Liaw, Tung-Wei Kao, Li-Wei Wu, Chung-Ching Wang, Hui-Fang Yang, Tao-Chun Peng, Yu-Shan Sun, Yaw-Wen Chang, Wei-Liang Chen

**Affiliations:** 1 Division of Family Medicine, Department of Family and Community Medicine, Tri-Service General Hospital and School of Medicine, National Defense Medical Center, Taipei, Taiwan(R.O.C); 2 Division of Geriatric Medicine, Department of Family and Community Medicine, Tri-Service General Hospital and School of Medicine, National Defense Medical Center, Taipei, Taiwan(R.O.C); 3 Graduate Institute of Medical Sciences, National Defense Medical Center, Taipei, Taiwan(R.O.C)

## Abstract

The direct relationship between metabolic syndrome (MetS) and function disability has not been established. The aim of the present study was to investigate the relationship between MetS and functional disability in the elderly. This retrospective observational study included 1,778 participants aged 60–84 years from the National Health and Nutrition Examination Survey (1999–2002). Impairments in activities of daily living (ADL), instrumental activities of daily living (IADL), leisure and social activities (LSA), lower extremity mobility (LEM), and general physical activities (GPA) were assessed. Additionally, the associations between the features of MetS and disability were evaluated. MetS was associated with a high prevalence of functional dependence in ADL, IADL, LSA, LEM, and GPA. After adjusting for potential confounders, a high number of MetS components was found to be associated with increased disability (P = 0.002). Additionally, associations were observed between MetS components, including abdominal obesity and high triglycerides levels, and functional dependence in ADL, IADL, LSA, LEM, and GPA (all, P < 0.05). A linear increase in disability might be associated with the number of MetS components in an elderly population. Additionally, MetS abnormalities, particularly abdominal obesity and high triglycerides levels, might be highly predictive of functional dependence in the elderly.

Over the last few decades, life expectancy has gradually increased in the developed countries. However, with the increase in life expectancy, the incidence of age-related disabilities and diseases has increased. Disability in the elderly population is a common reason for dependence, low quality of life, and the need for informal care. The functional decline in the elderly is expected to increase in the future^1^, and this will contribute to the unsustainable health care costs and will become a major public health problem. Underlying medical conditions and external factors determine the extent of physical impairment and the severity of disability. In the elderly, a reduction in functional decline and maintenance of physical capacity are required to preserve independent living.

Besides aging, metabolic syndrome (MetS) and the associated co-morbidities are considered to be significant healthcare problems in the elderly population^2^. MetS, defined as central obesity, glucose intolerance, atherogenic dyslipidemia, and high blood pressure, has been shown to be strongly associated with a high risk of coronary heart disease, cardiovascular disease, diabetes, and mortality^3^. The prevalence of MetS continues to increase in the elderly population in the US, and interventions for treating and preventing MetS would help avoid functional disability and enhance normal aging^4^.

The identification of potentially treatable risk factors of functional disability and early stage markers is critical for preventing the onset of disability in the growing elderly population. Understanding the characteristics of functional disability in the elderly may facilitate the development of interventions for slowing the rate of further decline. The direct relationship between functional disability and MetS has not been established. We hypothesized that the presence of a high number of features of MetS might be associated with functional disability in the elderly. The aim of the present study was to investigate the relationship between MetS and functional disability in the elderly, using data from the National Health and Nutrition Examination Survey (NHANES) from 1999 to 2002.

## Results

### Participants

A total of 21,004 participants were included in the NHANES dataset from 1999 to 2002. We excluded participants aged <60 years (N = 17,298) and aged ≥85 years (N = 409) to avoid age misclassification because all participants aged ≥85 were treated as aged 85 in NHANES 1999–2002. A total of 3,297 participants were aged between 60 and 84 years. We further excluded 1,519 persons with missing data, including disability questionnaire screening (N = 308), laboratory measurements (N = 251), clinical examinations (N = 960) were also excluded. Therefore, 1,778 participants were finally included in the present study.

### Characteristic of the study population

The characteristics of the participants stratified by MetS are summarized in Table 1. Among the 1,778 participants included in the present study, recruited in the study, the prevalence of MetS at baseline was 43%. In the MetS group, the mean age of the participants was 69.97 ± 6.93 years, and 41.9% of the participants were men. In the non-MetS group, the mean age of the participants was 70.23 ± 7.02 years, and 53.6% were men. Blood pressure, waist circumference (WC), self-reported dependence (activities of daily living [ADL], instrumental activities of daily living [IADL], leisure and social activities [LSA], lower extremity mobility [LEM], and general physical activities [GPA]), and serum levels of total cholesterol, triglycerides, glucose, and aspartate aminotransferase (AST) were significantly greater in the MetS group than in the non-MetS group (all, P < 0.05). However, the high-density lipoprotein cholesterol (HDL-C) levels and Digit Symbol Substitution Test(DSST) were significantly lower in the MetS group than in the non-MetS group (P < 0.001). Furthermore, the incidences of hypertension and diabetes mellitus, cognition impairment were higher in the MetS group than in the non-MetS group (both, p < 0.001).

### Metabolic components and functional dependence

The associations between the number of MetS components and the predicted total disability are presented in Table 2. In the adjusted analysis, the predicted total disability was higher in the MetS group than in the non-MetS group (P < 0.05). There was a strong linear increase in the predicted total disability with the increase in the number of MetS components. In the unadjusted analysis, the β coefficients of the predicted total disability considering 3, 4, and 5 MetS components were 0.446, 0.591, and 0.875 respectively (P-value for the trend <0.001). After additional adjustment, the β coefficient of the predicted total disability considering 5 MetS components was 0.573 (P-value for the trend <0.05). Moreover, hypertriglyceridemia and central obesity were significantly associated with higher predicted total disability in the fully adjusted models (P < 0.05).

The associations between the different metabolic components and each disability are presented in Table 3. After adjusting for covariates in Models 1–5, central obesity was found to be significantly associated with higher predicted ADL disability, IADL disability, LEM disability, and GPA disability (P < 0.05). High triglyceride levels were significantly associated with higher predicted ADL disability, IADL disability, LSA disability, and GPA disability (P < 0.05).

In table 4, there was a strong linear increase in predicted total disability with an increase in the number of MetS components in female elderly (p for trend <0.05), but not in male. In both women and men, abdominal obesity and hypertriglyceridemia was significantly associated with higher predicted total disability in fully adjusted models (p < 0.05).

## Discussion

The present study found that the risk of disability was significantly proportional to the number of MetS components. Additionally, abdominal obesity and high triglyceride levels were significantly associated with higher disability in the fully adjusted models. Very few studies have focused on the elderly population to investigate the association between MetS and disability^5^,^6^,^7^. To our knowledge, our study is the first to demonstrate that MetS is a potential risk factor for disability in the elderly US population.

Previous studies have shown that an increase in the rate of mobility dysfunction is associated with MetS^7^,^8^,^9^; however, the prevalence of MetS and definition of disability differed among the studies. Additionally, the majority of studies only focused on mobility limitations and MetS^8^,^9^. There is limited information on the association between the number of MetS components and the different domains of functional disability. Our findings of the association between MetS and function disability are consistent with the findings of previous studies in elderly people^7^,^8^,^9^. We found an association between the number of MetS components and total functional disability, as well as between the different MetS components and each domain of functional disability (ADL, IADL, LSA, LEM, and GPA).

In a 3-year longitudinal study of 1,155 elderly participants (the CHIANTI study), MetS was found to be associated with ADL disability^10^. The Health, Aging and Body Composition Study included 2,920 participants aged 70–79 years who were followed up for 4.5 years^8^. The study reported that MetS was associated with an adjusted relative risk (RR) of 1.46 (95% confidence interval [CI]= 1.30–1.63) for progression of mobility limitations, and the risk increased as the number of MetS components increased (P-value for the trend <0.001). In a cross-sectional study of 422 individuals living in 2 communities in southern Brazil^5^, MetS was found to significantly increase the risk of functional dependence, depression, and cognitive impairment. However, the number of participants was lower in that study than in the present study, and functional scales, such as LSA, LEM, and GPA, were not assessed. In a 3-year longitudinal study of 1,606 Mexican-Americans aged 60–98 years (the Sacramento Area Latino Study on Aging), MetS was found to be correlated with progressive limitations in mobility and high rates of impairment of ADL and IADL^6^. However, some data were missing owing to non-response and loss to follow-up, and this information was imputed through multiple imputation methodologies. In a 7-year longitudinal study of 6,141 French people aged >65 years, the presence of MetS was found to raise the risk of restricted mobility and IADL limitations^7^. However, a large number of participants were excluded owing to high rates of existing MetS and disability, and the exclusion likely weakened the observed associations rather than negated the results. Our study included a large population-based representative sample of older adults and provided further evidence for the association of each metabolic component with motion disability and daily living dysfunction. An increase in the number of MetS components was significantly associated with functional disability in the US adult population. It is important to determine the association of the number of MetS components with overall mobility dysfunction and the limitation in complex ADL, as limited information is available on the association between MetS and ADL/IADL^6^,^7^. To our knowledge, our study is the first to identify an association of MetS with LSA and GPA disability.

MetS has been shown to be a highly significant and independent predictor of the decline in mobility^7^,^9^. In an elderly population, MetS may be an obvious risk factor for functional disability. A previous study hypothesized that excessive free radicals and chronic inflammation are involved in the pathophysiological process of physical impairment^11^. High levels of C-reactive protein (CRP) and interleukin (IL)-6 have been shown to be associated with disability and low physical performance^12^, and the decline in physical function may be related to catabolic effects^13^. Kao *et al.* analyzed community-dwelling elderly people from the NHANES (1999–2002) and demonstrated that the serum total bilirubin levels within the normal range were inversely associated with the odds of functional dependence^14^. Serum bilirubin has been considered to have antioxidant and anti-inflammatory effects. Elevated homocysteine, which causes tissue injury by oxidative stress, has been shown to be associated with multiple domains of disability mediated by muscle strength and gait speed^15^. Previous studies have shown that patients with MetS have elevated levels of homocysteine^16^, CRP^17^, and proinflammatory mediators^18^. Total bilirubin levels have been shown to be inversely associated with MetS^19^. The cumulative effects of MetS components in the elderly population might result in chronic systemic inflammation that predisposes individuals to functional dependence and disability. The presence of MetS appears to worsen the age-related loss of serotoninergic innervation and responsiveness, leading to a high risk of depression^5^ and promoting increased functional dependence. In a prospective cohort study of 1,122 participants aged ≥71 years who were living in the community, lower extremity function was found to be a predictor of subsequent disability^20^. Previous studies have shown that muscle strength was inversely associated with the incidence of MetS^21^,^22^, and MetS might result in slow gait speed and related functional dependence.

The domains of IADL and LSA are associated with cognitive decline. MetS has been shown to be associated with cognitive decline and dementia^23^. Birdsill *et al.* found that low cerebral blood flow was associated with low memory function in patients with MetS^24^. Therefore, reducing the risk of MetS may be important for preserving cerebral blood flow and cognitive function. The presence of MetS has been shown to be associated with a low brain volume in patients with manifest arterial disease, even those without diabetes^25^. Cerebrovascular changes may weaken the frontal-subcortical circuit responsible for balance, gait, and movement.

In the present study, central obesity and high triglyceride levels were significantly associated with functional disability. These findings are consistent with previous findings and provide further information on the relationship between functional status and MetS. In a prospective study of 6,141 participants aged >65 years (the Three-City cohort study), central obesity and high triglyceride levels were found to be associated with limitations in mobility and IADL^7^. In an English longitudinal study of 3,055 community-dwelling adults aged ≥65 years, frailty was found to be associated with high abdominal circumference, even among underweight older participants^26^. A previous study showed that WC, a marker for central obesity, could predict the risk for most disability outcomes across the preretirement age period^27^. Studies have shown that abdominal adiposity is associated with high levels of biomarkers for oxidative stress^28^ and low-grade systemic inflammation^29^. Plasma leptin, secreted by adipocytes, has been shown to be associated with body fat mass in obese and lean participants^30^, and it may induce the production of IL-6, CRP, and other acute phase reactants. Plasma leptin has also been shown to exert an atherogenic effect by generating oxidative stress in endothelial cells^31^, and excessive oxidative stress has been shown to play an important role in the mechanisms of age-associated frailty^32^ and skeletal muscle damage^33^, leading to functional disability. We found a positive independent relationship between disability and MetS, which may be attributed to abdominal obesity mediated by insulin resistance. Further prospective studies are needed to determine the role of abdominal adiposity and insulin resistance in the relationship between disability and MetS.

High serum triglyceride levels were significantly associated with functional disability in the present study. Serum triglyceride is a clinically valuable marker of MetS. Elevated serum triglyceride levels were shown to be associated with insulin resistance^34^, and the presence of the insulin resistance-hyperinsulinemic-metabolic complex obstructs successful healthy ageing. A previous study showed that insulin resistance may be considered a biomarker of age-related diseases and reduced lifespan^35^. Moreover, the presence of insulin resistance may be considered a biomarker of susceptibility to mobility limitations among the elderly^36^. Furthermore, Cholerton *et al.*^37^ found that insulin resistance is associated with impaired cognitive function in the elderly. High triglyceride levels were associated with insulin resistance, which was related with cognition impairment and mobility limitation. Additionally, functional disability was associated with elevated serum triglyceride levels.

Our findings indicate that MetS and its individual components can be considered a cluster of risk factors for functional dependence, highlighting the increasing number of MetS components that are related to the worsening of physical disabilities. The present study has several strengths. The study included a community-based, racially mixed sample and discussed the effects of the number of MetS components and each MetS component on disabilities. In addition, multiple different instruments were used to evaluate physical function, leisure activity, and social activity. Furthermore, each MetS component and functional dependence domain was included for evaluation.

The present study has some limitations. First, this study was an observational, retrospective analysis of an existing database from a single period, rather than an analysis of long-term repeated observations with limited causal inferences. Second, the disability outcomes were self-reported, which may have led to over-reporting in participants who had metabolic disorders, such as obesity, or vascular disorders, such as hypertension. Third, data on potential confounding factors, such as IL-6, plasma leptin, and homocysteine, were not available in the NHANES datasets from 1999 to 2002. Forth, despite adjusting for a large number of potential confounding factors, residual confounding effects from unmeasured confounders of the association between MetS and disability might have been present. Fifth, we could not truly control for the diagnosed medical illness, as most information on medical history was self-reported. Finally, our sample was collected from 1999 to 2002. Therefore, the sample may not accurately reflect the current US population. However, based on a recent study on the prevalence of MetS in the US^38^, MetS continues to be common in older, non-Hispanic whites, which is consistent with our sample population.

## Conclusion

A linear increase in disability might be associated with the number of MetS components in an elderly population. It is important to evaluate the limitations in complex ADL, as well as mobility difficulties in the elderly. Additionally, MetS abnormalities, particularly abdominal obesity and high triglycerides levels, might be highly predictive of functional dependence in the elderly. Therefore, the prevention and treatment of MetS would be useful for promoting normal aging and preventing disability. A further prospective study is needed to establish the causality of MetS components in functional dependence. Our study provides epidemiologic evidence to support further studies on interventions for preventing disability in the elderly.

## Methods

### Study design and participants

The NHANES is a population-based health survey of non-institutionalized US citizens. It was performed by the National Center for Health Statistics (NCHS) of the Centers for Disease Control and Prevention (CDC). This was a continuous annual survey rather than a periodic survey. The NCHS performed physical examinations and an extensive household interview in a specially equipped mobile examination center. During the interview, trained examiners collected information on age, sex, race, medical history, the results of standard medical examinations, and the results of physical examinations. NHANES data has been released every 2 years since 1999. This data were extracted from the NHANES dataset from 1999 to 2002. Age is top-coded at 85 years in NHANES data releases for confidentiality reasons and all participants aged ≥85 were treated as aged 85. Based on previous studies^14^, we only enrolled individuals aged 60–84 to avoid age misclassification.

A total of 21,004 participants were included in the NHANES dataset from 1999 to 2002. We excluded participants aged <60 years (N = 17,298) and aged ≥85 years (N = 409). A total of 3,297 participants were aged between 60 and 84 years. We further excluded 1,519 persons with missing data, including disability questionnaire screening (N = 308), laboratory measurements (N = 251), missing data on metabolic syndrome (N = 960) were also excluded. Therefore, 1,778 participants were finally included in the present study.

The NHANES data can be downloaded from the NHANES website and analyzed without any permission. The Institutional Review Board of NCHS approved the NHANES, and our study did not require approval as it used de-identified data.

### Metabolic syndrome criteria

MetS was defined according to the revised definition of the National Cholesterol Education Program’s Adult Treatment Panel III (ATP III), which requires the presence of at least 3 of the following risk factors: (1) a high fasting glucose level (≥110 mg/dL) or use of anti-diabetic medication; (2) a low HDL-C level (<40 mg/dL in men or <50 mg/dL in women) or use of specific pharmacological treatment; (3) a high triglyceride level (≥150 mg/dL) or use of lipid-lowering medication; (4) central obesity, with a WC ≥102 cm in men or ≥88 cm in women; and (5) blood pressure ≥130/85 mmHg or use of anti-hypertensive medication^39^.

### Functional dependence

Nineteen questions on physical function were asked to evaluate the functional status of the participants. The questionnaire was designed to determine the condition of dependence, and an individual’s level of difficulty can be evaluated without using any special equipment. The questions were based on functional mobility and transfers, household productivity, manipulation of surroundings, and social integration. The 19 questions were divided into the following 5 domains: (1) ADL (eating, dressing, walking, and getting out of bed); (2) IADL (managing money, doing house chores, and preparing meals); (3) LSA (attending social events, going out to watch movies, and in-home leisure activities); (4)GPA (standing, bending, stooping, sitting, lifting, grasping, and reaching); and (5) LEM (walking one-quarter of a mile and walking up 10 steps). The following 4 levels of difficulty were used to evaluate each domain: no difficulty, some difficulty, much difficulty, and unable to perform. Total disability was assessed as the sum of the 5 major domainsat baseline and analyzed as a continuous variable, with a range from 0 to 19. Furthermore, we defined each disability as difficulty performing one or more activities within the domainand analyzed the domains as continuous variables, with ranges from 0 to 4 (ADL), 0 to 3 (IADL), 0 to 3 (LSA), 0 to 2 (LEM), and 0 to 7 (GPA).

### Covariates

The self-reported data included age, sex, race/ethnicity, smoking history, and medical history. Participants were asked the question “Do you now smoke cigarettes?” to determine the smoking status. Participants were considered to have heart disease if they had been diagnosed with the condition or if they experienced myocardial infarction, coronary artery disease, angina, or congestive heart failure. Stroke was self-reported. Participants were considered to have diabetes if they had been diagnosed with the condition by a doctor, if they were using anti-diabetic medication, or if the fasting glucose level was ≥126 mg/dL or the random glucose level was ≥200 mg/dL. Blood pressure was measured 3 to 4 times using a mercury sphygmomanometer in the right arm unless the participant has a specific condition, and the mean systolic and diastolic blood pressure values were recorded. Participants were considered to have hypertension if the mean blood pressure was ≥140/90 mmHg, if they were using anti-hypertensive medication, or if they had been diagnosed with the condition by a doctor. WC was measured to the nearest 0.1 cm at the high point of the iliac crest during minimal respiration. Digit Symbol Substitution Test (DSST), a subtest of the Wechsler Adult Intelligence Scale, can examine the visuospatial and motor speed of processing. DSST is a nonverbal test that evaluates psychomotor speed and represents a sensitivemeasure of frontal lobe executive functions. In DSST coding exercise, participants transcribe symbols matched to numbers using a legend. The score represented the number of correct items completed within period of 120 seconds and the maximum score is 133. Because of the absence of agree-upon threshold for normal DSST score^40^, we categorized the DSST score into four groups. The quartiles of DSST score were as follows: Q1 ≦ 28, 28 < Q2 ≦ 40, 40 < Q3 ≦ 53, and Q4 > 53. The lowest quartile (Q1) of DSST score is considered as cognition impairment.

The levels of total cholesterol, triglycerides, HDL-C, AST, and alanine aminotransferase were measured enzymatically using the Hitachi-704 analyzer (Boehringer Mannheim Diagnostics, Indianapolis, IN). Low-density lipoprotein cholesterol levels were calculated using the Friedewald formula^41^. Total bilirubin levels were assessed using automated biochemical profiling (Beckman Synchron LX20; Beckman Coulter Inc., Fullerton, CA), and serum glucose levels were measured using an enzymatic assay (Cobas Mira assay; Roche, Basel, Switzerland). Additionally, CRP levels were determined using a highly sensitive assay technique and were quantified with latex-enhanced nephelometry using a Behring Nephelometer System (Dade Behring, Deerfield, IL).

All the protocols used standardized methods with documented accuracy with respect to CDC reference methods. All detailed specimen collection information is available on the NHANES website.

### Statistical analysis

The NHANES data sets from 1999 to 2000 and 2001 to 2002 were combined according to the NHANES analytic guidelines. The predicted values of self-reported dependence were divided into MetS and non-MetS groups. The Chi-square test was used to analyze categorical data, and the Mann-Whitney *U* test was used to analyze continuous data. The associations between self-report dependence and the number of MetS components or each individual MetS component were determined. From a clinical standpoint, demographic factors can influence the results; therefore, they were used in covariate adjustment. Five multiple linear regression models were constructed, and of these, 1 was unadjusted and the other 4 were adjusted. Model 1 was unadjusted. Model 2 included Model 1+ age, sex, and race/ethnicity adjustment. Model 3 included Model 2+ serum AST, serum total cholesterol, and serum total bilirubin adjustment. Model 4 included Model 3+ smoking, hypertension, type 2 diabetes mellitus, and stroke adjustment. Model 5 included Model 4 + cognition impairment adjustment. Moreover, we also evaluated effect on the association with MetS and disability in gender categorization. The P-values for the trend tests were determined by treating the number of MetS components as a continuous variable (1–5) in order to observe the associations between an increase in the number of MetS components and self-reported dependence. All statistical analyses were performed using SPSS (version 18.0 for Windows; IBM, Armonk, NY). Two-sided P-values <0.05 were considered to indicate significant differences.

## Additional Information

**How to cite this article**: Liaw, F.-Y. *et al.* Components of Metabolic Syndrome and the Risk of Disability among the Elderly Population. *Sci. Rep.* **6**, 22750; doi: 10.1038/srep22750 (2016).

## Figures and Tables

**Table 1:** Participants characteristics with or without metabolic syndrome.

Variables	N = 1778		
	Non-metabolic syndrome N = 1004	Metabolic syndrome N = 774	*P*value
Continuous variables			
Age (years), mean (SD)	70.23(7.02)	69.97(6.93)	0.451
Systolic blood pressure, mean (SD)	133.67(20.93)	145.44(20.69)	<0.001
Diastolic blood pressure, mean (SD)	68.83(12.28)	72.81(13.80)	<0.001
Waist circumference (cm), mean (SD)	95.61(12.48)	105.38(11.75)	<0.001
Serum total cholesterol (mg/dL), mean (SD)	205.35(36.43)	212.19(43.05)	<0.001
Serum triglycerides (mg/dL), mean (SD)	115.43(54.18)	201.10(135.88)	<0.001
LDL (mg/dL), mean (SD)	130.30(33.35)	127.77(36.39)	0.303
HDL (mg/dL), mean (SD)	57.52(15.88)	46.56(14.25)	<0.001
C-reactive protein (mg/dL), mean (SD)	0.47(0.84)	0.51(0.70)	0.324
Serum albumin (g/dL), mean (SD)	4.30(0.28)	4.28(0.28)	0.209
Serum AST (U/L), mean (SD)	22.92(4.82)	21.98(5.03)	<0.001
Serum total bilirubin (umol/L), mean (SD)	11.49(4.61)	10.78(4.02)	0.001
Serum glucose (mg/dL), mean (SD)	95.35(25.79)	115.11(43.17)	<0.001
DSST score	45.44(18.671)	41.84(18.358)	<0.001
Self-reported dependence			
ADL, *n* (%)	28(2.8)	31(4.0)	0.156
IADL, *n* (%)	51(5.1)	64(8.3)	0.007
LSA, *n* (%)	40(4.0)	47(6.1)	0.043
LEM, *n* (%)	83(8.6)	99(13.4)	0.001
GPA, *n* (%)	223(22.2)	234(30.2)	<0.001
Categorical variables ^c^			
Men, n (%)	528(53.6)	324(41.9)	<0.001
Non-Hispanic white, *n* (%)	641(63.8)	442(57.1)	0.004
Hypertension, *n* (%)	584(58.2)	635(82.0)	<0.001
Diabetes mellitus, *n* (%)	102(10.2)	217(28.0)	<0.001
Stroke, *n* (%)	46(4.6)	44(5.7)	0.291
Heart disease, *n* (%)	164(16.3)	148(19.1)	0.126
Smoke, *n* (%)	128(12.7)	81(10.5)	0.138
Cognition impairment*, *n* (%)	195(19.4)	191(24.6)	0.005

BMI, body mass index; LDL, low-density lipoprotein; HDL, high-density lipoprotein; AST, aspartate aminotransferase; ADL, activities of daily living; IADL, instrumental activities of daily living; LSA, leisure and social activities; LEM, lower extremity mobility; GPA, general physical activities; CAD, coronary artery disease; SD, standard deviation.

^*^Cognition impairment was defined as the lowest quartile of DSST score.

**Table 2:** Regression coefficients of presence and number of metabolic syndrome for elderly patients with total disability*.

Variables	Model 1		Model 2		Model 3		Model 4		Model 5	
	*β* (95% CI)	*P* value	*β* (95% CI)	*P*value	*β* (95% CI)	*P* value	*β* (95% CI)	*P* value	*β* (95% CI)	*P* value
Presence of metabolic syndrome	0.366 (0.170, 0.562)	<0.001	0.306 (0.109, 0.503)	0.002	0.283 (0.085, 0.480)	0.005	0.219 (0.012, 0.426)	0.038	0.216 (0.011,0.422)	0.039
Number of metabolic syndrome components										
1	0.073 (−0.315, 0.460)	0.712	0.021 (−0.364, 0.406)	0.915	−0.007 (−0.391, 0.377)	0.973	0.077 (−0.309, 0.463)	0.696	0.058 (−0.325,0.441)	0.767
2	0.362 (−0.010, 0.734)	0.057	0.328 (−0.041, 0.698)	0.082	0.29 (−0.079, 0.658)	0.124	0.351 (−0.026, 0.729)	0.068	0.327 (−0.048,0.702)	0.087
3	0.446 (0.063, 0.829)	0.022	0.359 (−0.023, 0.741)	0.065	0.322 (−0.060, 0.703)	0.098	0.380 (−0.017, 0.776)	0.060	0.362 (−0.031,0.755)	0.071
4	0.591 (0.184, 0.999)	0.004	0.503 (0.096, 0.909)	0.016	0.44 (0.032, 0.848)	0.034	0.421 (−0.007, 0.849)	0.054	0.411 (−0.014,0.835)	0.058
5	0.875 (0.346, 1.404)	0.001	0.739 (0.211, 1.267)	0.006	0.681 (0.154, 1.208)	0.011	0.632 (0.079, 1.186)	0.025	0.573 (0.024,1.123)	0.041
*P* for trend	<0.001		<0.001		<0.001		0.002		0.002	
Components of metabolic syndrome										
Abdominal obesity	0.495 (0.293, 0.697)	<0.001	0.458 (0.251, 0.664)	<0.001	0.446 (0.240, 0.651)	<0.001	0.451 (0.243, 0.658)	<0.001	0.447 (0.241,0.653)	<0.001
High blood pressure	0.002 (−0.147, 0.151)	0.977	−0.039 (−0.187, 0.110)	0.610	−0.044 (−0.192, 0.105)	0.564	−0.05 (−0.218, 0.119)	0.563	−0.08 (−0.248,0.087)	0.348
High triglycerides	0.413 (0.208, 0.618)	<0.001	0. 38 (0.175, 0.585)	<0.001	0.385 (0.177, 0.593)	<0.001	0.317 (0.108,0.526)	0.003	0.36 (0.153, 0.567)	0.001
Low HDL−cholesterol	0.301 (0.089, 0.514)	0.005	0.285 (0.073, 0.497)	0.008	0.231 (0.018, 0.443)	0.034	0.17 (−0.042,0.381)	0.115	0.163 (−0.047, 0.372)	0.128
High glucose	0.3 (0.092, 0.508)	0.005	0.313 (0.106, 0.520)	0.003	0.265 (0.058, 0.472)	0.012	0.112 (−0.111,0.335)	0.324	0.074 (−0.148, 0.295)	0.513

Model 1 = unadjusted Model 2 = Model 1 + age, gender, and race−ethnicity Model 3 = Model 2 + (serum AST, serum total cholesterol, and serum total bilirubin) Model 4 = Model 3 + (smoking, hypertension, type 2 diabetes mellitus, and stroke) Model 5 = Model 4 + (Cognition impairment#).

^#^Cognition impairment was defined as the lowest quartile of DSST score.

^*^Total disability was defined as any difficulty in performing one or more activities within a given domain and assessed as the sum of five major domains at baseline and analyzed as a continuous variable.

**Table 3:** Regression coefficients of the component of metabolic syndrome for elderly patients with eachdomain of disability.

Variables		ADL Disability		IADL Disability		LSA Disability		LEM Disability		GPA Disability	
		β (95% CI)	P value	β (95% CI)	P value	β (95% CI)	P value	β (95% CI)	P value	β (95% CI)	P value
Model 1	Abdominal obesity	0.041(0.012, 0.069)	0.005	0.061(0.027, 0.095)	<0.001	0.026(−0.012, 0.063)	0.181	0.092(0.047, 0.136)	<0.001	0.283(0.168, 0.397)	<0.001
	High blood pressure	0.004(−0.017, 0.025)	0.724	0.01(−0.015, 0.035)	0.435	0.002(−0.026, 0.029)	0.915	−0.006(−0.039, 0.026)	0.698	−0.007(−0.091, 0.077)	0.871
	High triglycerides	0.04(0.010, 0.069)	0.008	0.061(0.026, 0.096)	0.001	0.069(0.031, 0.107)	<0.001	0.031(−0.015, 0.076)	0.183	0.217(0.104, 0.330)	<0.001
	Low HDL−cholesterol	0.039(0.009, 0.069)	0.012	0.013(−0.024, 0.050)	0.487	0.03(−0.009, 0.069)	0.133	0.051(0.004, 0.098)	0.032	0.172(0.055, 0.289)	0.004
	High glucose	0.023(−0.006, 0.053)	0.121	0.027(−0.009, 0.063)	0.138	0.043(0.005, 0.082)	0.029	0.054(0.008, 0.099)	0.021	0.16(0.046, 0.275)	0.006
Model 2	Abdominal obesity	0.039(0.010, 0.068)	0.009	0.059(0.024, 0.094)	0.001	0.025(−0.013, 0.064)	0.199	0.086(0.040, 0.131)	<0.001	0.256(0.139, 0.372)	<0.001
	High blood pressure	0.001(−0.020, 0.022)	0.920	0.005(−0.020, 0.031)	0.680	−0.003(−0.030, 0.025)	0.852	−0.014(−0.047, 0.018)	0.388	−0.028(−0.112, 0.056)	0.509
	High triglycerides	0.034(0.005, 0.064)	0.022	0.059(0.023, 0.094)	0.001	0.065(0.026, 0.103)	0.001	0.031(−0.014, 0.076)	0.177	0.195(0.082, 0.308)	0.001
	Low HDL−cholesterol	0.035(0.005, 0.065)	0.024	0.012(−0.025, 0.049)	0.525	0.027(−0.012, 0.067)	0.172	0.053(0.006, 0.099)	0.027	0.16(0.044, 0.277)	0.007
	High glucose	0.023(−0.007, 0.053)	0.128	0.027(−0.009, 0.063)	0.141	0.041(0.002, 0.080)	0.038	0.061(0.015, 0.107)	0.009	0.17(0.056, 0.284)	0.003
Model 3	Abdominal obesity	0.038(0.009, 0.067)	0.011	0.057(0.022, 0.092)	0.002	0.023(−0.016, 0.061)	0.249	0.086(0.040, 0.131)	<0.001	0.25(0.134, 0.367)	<0.001
	High blood pressure	0.001(−0.020, 0.022)	0.903	0.004(−0.021, 0.029)	0.768	−0.003(−0.031, 0.024)	0.811	−0.014(−0.046, 0.019)	0.418	−0.032(−0.116, 0.052)	0.453
	High triglycerides	0.037(0.007, 0.067)	0.016	0.056(0.019, 0.092)	0.003	0.064(0.025, 0.103)	0.001	0.036(−0.010, 0.082)	0.129	0.198(0.083, 0.313)	0.001
	Low HDL-cholesterol	0.03(−0.001, 0.060)	0.057	0.005(−0.032, 0.042)	0.780	0.02(−0.020, 0.060)	0.319	0.043(−0.004, 0.090)	0.074	0.134(0.017, 0.251)	0.025
	High glucose	0.019(−0.011, 0.049)	0.216	0.019(-0.017, 0.055)	0.290	0.033(−0.006, 0.072)	0.095	0.055(0.009, 0.101)	0.019	0.148(0.033, 0.262)	0.011
Model 4	Abdominal obesity	0.043(0.013, 0.073)	0.004	0.058(0.023, 0.094)	0.001	0.027(−0.012, 0.066)	0.171	0.085(0.039, 0.131)	<0.001	0.245(0.127, 0.363)	<0.001
	High blood pressure	0.009(−0.015, 0.033)	0.439	0.007(−0.022, 0.035)	0.652	0.001(−0.030, 0.033)	0.933	−0.028(−0.066, 0.009)	0.143	−0.041(−0.137, 0.055)	0.401
	High triglycerides	0.033(0.003, 0.063)	0.033	0.049(0.012, 0.085)	0.009	0.058(0.018, 0.097)	0.004	0.026(−0.021, 0.072)	0.275	0.156(0.041, 0.272)	0.008
	Low HDL−cholesterol	0.026(−0.005, 0.057)	0.098	−0.001(−0.038, 0.036)	0.969	0.013(−0.026, 0.053)	0.515	0.035(−0.012, 0.082)	0.146	0.097(−0.020, 0.214)	0.103
	High glucose	0.009(−0.024, 0.041)	0.590	0.006(−0.033, 0.045)	0.753	0.018(−0.024, 0.061)	0.395	0.037(−0.013, 0.087)	0.147	0.05(−0.074, 0.173)	0.430
Model 5	Abdominal obesity	0.043(0.013, 0.072)	0.005	0.058(0.022, 0.093)	0.001	0.027(−0.012, 0.066)	0.177	0.085(0.039, 0.131)	<0.001	0.243(0.126,0.36)	<0.001
	High blood pressure	0.07(−0.017, 0.031)	0.571	0.001(−0.028,0.029)	0.962	−0.002(−0.034, 0.029)	0.879	−0.031(−0.068, 0.007)	0.111	−0.058(−0.1539, 0.038)	0.236
	High triglycerides	0.037(0.007, 0.067)	0.017	0.058(0.022, 0.094)	0.002	0.063(0.023, 0.102)	0.002	0.03(−0.017, 0.076)	0.208	0.178(0.063,0.293)	0.002
	Low HDL−cholesterol	0.025(−0.005, 0.056)	0.106	−0.002(−0.039, 0.034)	0.905	0.012(−0.027, 0.052)	0.541	0.034(−0.013, 0.081)	0.156	0.094(−0.022, 0.21)	0.114
	High glucose	0.005(−0.027, 0.038)	0.747	−0.002(−0.04, 0.037)	0.933	0.014(−0.028, 0.056)	0.515	0.033(−0.017, 0.083)	0.19	0.03(−0.093, 0.153)	0.63

Model 1 = unadjusted Model 2 = Model 1 + age, gender, and race-ethnicity Model 3 = Model 2 + (serum AST, serum total cholesterol, and serum total bilirubin) Model 4 = Model 3 + (smoking, hypertension, type 2 diabetes mellitus, and stroke) Model 5 = Model 4 + (Cognition impairment*) Each disability was defined as any difficulty in performing one or more activities within a given domain and assessed as the sum of each major domain at baseline and analyzed as a continuous variable.

^*^Cognition impairment was defined as the lowest quartile of DSST score.

**Table 4:** Regression coefficients of presence and number of metabolic syndrome for elderly patients with total disability according to gender.

Variables	Men(N = 862)				Women(N = 916)			
	Unadjusted		Full adjusted		Unadjusted		Full adjusted	
	β (95% CI)	P value	β (95% CI)	P value	β (95% CI)	P value	β (95% CI)	P value
Presence of metabolic syndrome	0.344(0.083, 0.605)	0.010	0.195(−0.071, 0.461)	0.151	0.316(0.024, 0.608)	0.034	0.237(−.075, 0.55)	0.136
Number of metabolic syndrome components								
1	−0.026(−0.495, 0.442)	0.913	−0.026(−0.491, 0.439)	0.912	0.195(−0.437, 0.828)	0545	0.098(−0.527, 0.723)	0.758
2	0.05(−0.398, 0.498)	0.825	−0.016(−0.468, 0.436)	0.946	0.725(0.115, 1.335)	0.02	0.729(0.113, 1.345)	0.02
3	0.141(−0.335, 0.616)	0.562	0.057(−0.426, 0.54)	0.816	0.718(0.104, 1.333)	0.022	0.678(0.043, 1.313)	0.036
≧4	0.526(0.031, 1.022)	0.037	0.254(−0.265, 0.774)	0.337	0.786(0.175, 1.398)	0.012	0.681(0.032, 1.329)	0.04
P for trend	0.003		0.11		0.001		0.008	
Components of metabolic syndrome								
Abdominal obesity	0.395(0.142, 0.648)	0.002	0.425(0.176, 0.675)	0.001	0.479(0.146, 0.813)	0.005	0.464(0.127, 0.801)	0.007
High blood pressure	−0.072(−0.259, 0.115)	0.448	−0.205(−0.409, −0.001)	0.049	0.039(−0.195, 0.273)	0.743	0.103(−0.169, 0.375)	0.457
High triglycerides	0.412(0.137, 0.687)	0.003	0.306(0.031, 0.581)	0.029	0.372(0.07 0.674)	0.016	0.391(0.84, 0.698)	0.013
Low HDL cholesterol	0.337(0.054, 0.620)	0.02	0.205(−0.072, 0.482)	0.146	0.251(−0.063, 0.564)	0.117	0.117(−0.195, 0.429)	0.463
High glucose	0.295(0.028, 0.562)	0.031	0.052(−0.226, 0.330)	0.712	0.39(0.07, 0.71)	0.017	0.089(−0.257, 0.436)	0.613

Full adjust = Age, race-ethnicity, serum AST, serum total cholesterol, serum total bilirubin, smoking, hypertension, type 2 diabetes mellitus, stroke and cognition impairment. Cognition impairment was defined as the lowest quartile of DSST score.
